# The impact of accompaniment on the training and competencies of psychology students for clinical practice: a quasi-experimental study

**DOI:** 10.3389/fpsyg.2025.1657514

**Published:** 2026-01-05

**Authors:** Karla Gallo-Giunzioni, Agata Kasprzak, Elvira Santiago

**Affiliations:** Faculty of Education and Psychology, Universidad Francisco de Vitoria, Madrid, Spain

**Keywords:** accompaniment, clinical supervision, competencies, educational innovation, psychology

## Abstract

**Introduction:**

Training in psychology requires not only theoretical knowledge but also the development of practical competencies for intervention in healthcare settings. This study aimed to evaluate the effectiveness of a teaching innovation program based on simulated contexts under the accompaniment and supervision of a qualified instructor, designed to strengthen competencies essential for future professional practice.

**Methods:**

A program was implemented based on the practical application of intervention and assessment tools in simulated clinical settings. A pre-post design was used with 135 third-year psychology students over two academic years (2023–2024 and 2024–2025), of whom 111 were women (82.2%) and 24 men (17.8%) (N_2023−2024_ = 68; N_2024−2025_ = 67) that participated in five simulated clinical sessions. The program's impact was assessed using the COMPES Self-Report Questionnaire, evaluating competencies such as teamwork, autonomous learning, problem-solving, communication, and ethical awareness.

**Results:**

The results showed significant improvements in all evaluated competencies except for teamwork, possibly due to the limited duration of the program and minimal collaborative interaction among participants.

**Discussion:**

Expert supervision and accompaniment appear to be key factors in competence development. This innovative approach reinforces the integration of theory and practice in psychological training and offers a replicable methodology to optimize experiential learning.

## Introduction

The current professional environments demand mastery of both technical and professional competencies—transferable skills that are applicable in diverse contexts and highly sought after across socio-occupational contexts (Martínez Clares et al., 2018). Higher education institutions face a considerable challenge, as the current educational landscape goes beyond the mere acquisition of theoretical knowledge. Accordingly, universities must be oriented toward the development of practical skills and specific professional attitudes (Carraccio et al., 2016).

Unlike other disciplines, psychology requires students to master not only conceptual frameworks but also practical competencies for future work with diverse human experiences. This formative demand is not limited to theoretical understanding; it involves preparing students for responsible and ethical practice. Psychological training must therefore foster the development of skills that enable the establishment of an effective therapeutic relationship in which professionals not only apply knowledge but also act as facilitators of personal growth (Ruiz Rodríguez et al., 2018). Accompaniment, understood as the task of guiding patients through their personal development process, requires specific dispositions, attitudes, and competencies associated with the health field (Fruggeri et al., 2023).

Dispositions refer to innate capacities, such as empathy or active listening, that orient the therapist toward an effective accompaniment (Kiesler et al., 2023). Attitudes are those that direct the will to act during the therapeutic process, such as respecting the patient's timing or maintaining a non-judgmental stance. Competencies, finally, are understood as a set of acquired and trainable habits that empower professionals to face the demands of a given issue comprehensively, including emotional self-regulation (Prieto, 2011; Benítez Hernández et al., 2021).

Various studies (Martínez Clares et al., 2018; Benítez Hernández et al., 2021; Llanes Ordóñez et al., 2017) in higher education emphasize the importance of placing the student at the center of their professional training, as responsible agents in articulating the skills acquired to solve real-world problems. This reality calls for a curricular transition toward competency-based education in the health field (Herrmann-Werner et al., 2017).

Spanish universities promote innovative methodologies and formative assessment to bring students closer to professional reality (Benítez Hernández et al., 2021). However, learning to accompany without having been accompanied represents a challenge for future psychologists. Consequently, curricular models emphasize pedagogical strategies that expose students to real contexts, fostering autonomy and responsibility (Camerino et al., 2019; Greenberg and Minter, 2019; Maldonado et al., 2022). Literature supports that active learning improves both practical application and student satisfaction, although it requires attention to the ethical and legal responsibilities of educators and professionals (Benítez Hernández et al., 2021).

Practical activity in psychology training has become a core factor in the acquisition and consolidation of specific competencies for psychologists, including assessment, intervention, and human accompaniment throughout the psychotherapeutic process. The university classroom is, therefore, students' first approach to clinical reality. Hence, university teaching must be oriented toward accompanying students in acquiring the relevant competencies that will enable them to grow and mature both professionally and personally, thus ensuring the quality and effectiveness of their future professional practice (García-Pérez and Mendía, 2015).

Psychology academics face the complex task of teaching how to accompany, a competency that goes beyond theoretical acquisition and delves into managing the therapeutic relationship (Prado-Abril et al., 2017). Scientific studies and the implementation of pedagogical methodologies with more practical content in university settings have shown that strengthening these basic competencies is a major challenge, although results are promising (Benítez Hernández et al., 2021; Maldonado et al., 2022). One of the main obstacles identified by research lies in the prevalence of traditional teaching methodologies characterized by lectures and repetition, which undermine reflective analysis by students (Rodríguez Gómez et al., 2017).

Implementing innovative methodologies demands a transformation in the tasks and expectations of the teaching role to supervise the development of necessary competencies in students (Carraccio et al., 2016). A competency-based training approach requires educators to adopt an active role in ethically and effectively guiding future professionals in their initial encounters with clinical realities (American Psychological Association, 2015).

In professional practice, this approach is known as clinical supervision and is considered a fundamental process in the training of health psychologists (He et al., 2022). Supervision involves reflection and guidance by a more experienced specialist who supports less experienced psychologists in facing complex situations, achieving greater objectivity, enriching hypotheses, expanding techniques and approaches, and deepening technical, emotional, and cultural difficulties that may influence the therapeutic alliance and the therapy's progress (American Psychological Association, 2015; Kangos et al., 2018). This process strengthens therapists' self-efficacy and self-awareness, allowing them to address clinical cases with greater confidence and precision (Lohani and Sharma, 2023).

In a systematic review, it was found that clinical supervision significantly affects burnout, the well-being of health professionals, and job satisfaction (Martin et al., 2021). Psychology students, who are in the early stages of their training, require qualified educators to provide a higher level of structure and guidance throughout the learning process, along with positive feedback, validation of their observations, alternative proposals, precise guidance, and access to relevant bibliographic resources (Hidalgo-Cobo, 2024).

Therapists go through various phases in their professional development, each requiring specific competencies from the supervisor (Martín and Fonseca, 2017). The supervisory space is designed to support professional development by fostering reflection on technical and ethical aspects, and personal growth by providing a safe space to explore individual resonances, promoting ethical responsibility (Yung, 2023). It is important to note that supervision offers a space to address students' personal difficulties and evaluate their impact on future clinical practice (Yung, 2023). However, due to psychotherapeutic limitations, personal therapy is recommended to develop skills beyond the scope of supervision (He et al., 2022).

Clinical supervision and accompaniment in psychotherapy share a formative and supportive purpose for professionals in training; however, they differ in their professional aims. Scientific literature characterizes clinical supervision as a formal, structured, and evaluative process, guided by an experienced professional, whose objective is to foster the development of clinical competencies, review professional practice, and ensure the quality of the services provided (American Psychological Association, 2015; He et al., 2022; Kangos et al., 2018). This process maintains clear boundaries with psychotherapy, as its purpose is educational rather than therapeutic.

In contrast, accompaniment is conceived as a horizontal and dialogical practice, less theoretically formalized, based on collaboration among peers. Its aim is to provide emotional support, encourage joint reflection, and humanize professional practice, without implying a hierarchical or evaluative relationship (Silva and Sousa, 2018). Accompaniment represents an ethical and empathetic form of intervention that complements—but does not replace—clinical supervision in the formative processes of psychology trainees (Silva and Sousa, 2018; Follette and Batten, 2000).

The relevance of accompaniment and psychological supervision, along with the growing interest in strengthening the practical development of competencies in future health professionals, underscores the need to incorporate these experiences into university curricula (Martín and Fonseca, 2017; Crespí et al., 2022; Gil-Galván et al., 2020). Nevertheless, the acquisition of essential competencies for therapeutic accompaniment is limited when relying exclusively on traditional methodologies. In response, innovative approaches such as problem-based learning and cooperative learning are highlighted for promoting student participation and optimizing competency assessment (Fernández-Jiménez et al., 2014).

Problem-based learning is a student-centered methodology involving research and reflection on a classroom-presented problem, allowing students to acquire and apply knowledge for its resolution, whether the scenario is real or fictional (Carrasco-Huamán, 2022). This methodology clearly supports the development of competencies transferable to the professional domain, demonstrating its usefulness in the university learning context. Cooperative learning, on the other hand, focuses on training social skills necessary for teamwork and essential for personal and professional functioning. This teaching methodology promotes reflective learning and student autonomy, requiring them to work collaboratively, take active responsibility for task execution, resolve interpersonal conflicts, and improve academic performance (Carrasco-Huamán, 2022).

Ten core competencies have been identified for psychology students, demanding active learning management and the development of skills applicable both inside and outside academic settings (Gómez-Ruiz et al., 2013). These include: (a) knowledge application, (b) argumentation, (c) problem-solving, (d) information analysis, (e) communication, (f) autonomous learning, (g) ethical awareness, (h) creativity, (i) teamwork, and (j) evaluation. These competencies are transferable to other contexts and depend on students' professional and personal maturation (Gómez-Ruiz et al., 2013).

Since these competencies contribute not only to academic training but also to the comprehensive preparation of students for real clinical practice, it is essential to design learning experiences that foster them in an integrated manner. Therefore, this study proposes the implementation of an educational innovation project focused on developing key therapeutic competencies in psychology students through the practical application of intervention and assessment tools in supervised and simulated contexts. In addition, it seeks to evaluate the impact of the program through student observation and faculty accompaniment of a clinical case represented by a qualified actor and observed through a one-way mirror, allowing for direct and reflective interaction with real clinical-ethical conflicts in a controlled environment. Consequently, the present research aims to address the following question: To what extent does an educational innovation program based on accompaniment and supervised practice contribute to the development of competencies in psychology students?

## Materials and methods

### Study design and participants

The present study used a quasi-experimental design with pre- and post-intervention measures, applied to a sample collected during the 2023–2024 and 2024–2025 academic cycles.

The analysis included 135 third-year Psychology students from two academic cohorts, aged between 19 and 29 years. Table 1 presents the sociodemographic characteristics of the participants.

**Table 1:** Sociodemographic characteristics of the sample (*N* = 135).

**Variable**	***N***	**%**	**M (SD)**
**Gender**			
Male	24	17.8	
Female	111	82.2	
Age			20.88 (1.43)
**Academic year**			
2023–2024	68	50.4	
2024–2025	67	49.6	

M, Mean; SD, standard deviation.

The groups were formed with students enrolled in the aforementioned courses. Each group consisted of 7–9 students. The groups organized for the course Clinical Framework also functioned for Models, so separate groups were not created for each course. In cases where a student was not enrolled in one of the two courses, their grade was not affected in the course in which they were enrolled. In total, five groups were constituted, each assigned to a practical session.

### Instruments

Sociodemographic Questionnaire. An *ad hoc* instrument collecting data on age, sex, and academic cohort within the Psychology degree program.

COMPES-Self-Report Questionnaire on Basic Competencies in University Student Assessment (Gómez-Ruiz et al., 2013). This instrument enables participants to self-assess their performance across 37 different academic scenarios related to 10 fundamental competencies in the evaluation process. These competencies include: (1) knowledge application, (2) argumentation, (3) problem-solving, (4) information analysis, (5) communication, (6) autonomous learning, (7) ethical awareness, (8) creativity, (9) teamwork, and (10) assessment. The instrument demonstrated adequate Cronbach's alpha in its original version (α = 0.94) and in this study α = 0.91 in the pre-evaluation and α = 0.94 in the post-evaluation.

### Procedure

During the 2023–2024 and 2024–2025 academic years, within the curriculum of the courses Models, Techniques and Instruments for Assessment and Psychodiagnosis and Clinical Framework and Therapeutic Encounter, this study was conducted as part of the Innova teaching innovation project, approved in July 2023 by the Academic Commission of the Francisco de Vitoria University (Madrid).

The project involved the planning and organization of student groups who participated in the preparation and application of five sessions with a simulated patient. Students received preliminary case information (patient's name and age). The practical sessions were distributed between the two courses: Sessions 1, 2, and 5 corresponded to Clinical Framework and Therapeutic Encounter, while Sessions 3 and 4 were conducted within Models, Techniques and Instruments for Assessment and Psychodiagnosis (see Table 2).

**Table 2:** Summary of the innovation programme.

**Session no**.	**Course**	**Objectives**	**Session outline**	**Post-session contents**
1	Clinical framework and therapeutic encounter	Explore the reason for consultation, gather the patient's emotional state, and establish guidelines for the sessions.	- First meeting with the patient. - Establishment of the session framework (schedules, fees, frequency, duration, payment method, etc.).	-Initial understanding of the reason for the consultation. -Establishing a framework. -Formulating possible hypotheses. -Identifying students' personal experiences brought up by the case.
2	Clinical framework and therapeutic encounter	Explore in detail the areas of patient functioning related to their reason for consultation.	- Case exploration. - Introduction to the evaluation for the next session. - Clarification of doubts about the evaluation process.	-Formulation of explanatory hypotheses. -Selection of assessment tools. -Identification of students' personal experiences and potential future challenges.
3	Models, techniques, and instruments of evaluation and psychodiagnosis	Apply evaluation techniques and prepare a report integrating the information obtained.	- Administration of psychometric and projective evaluation tests.	-Collection and integration of patient information. -Proofreading and preparation of written reports.
4	Models, techniques, and instruments of evaluation and psychodiagnosis	Provide oral feedback of the report, address the patient's doubts, and propose a therapeutic resource.	- Presentation of the report to the patient. - Addressing doubts and concerns. - Proposal of a therapeutic resource.	-Proposal for a therapeutic plan
5	Clinical framework and therapeutic encounter	Close the psychotherapeutic process and manage emotions associated with termination.	- Final feedback on the therapeutic process. - Emotional containment and closure.	-Conclusion of the therapeutic process. -Appropriate management of closure and its emotional implications. -Students' personal reflection on the impact of their emotional interferences.

Students were informed in advance that all materials and information generated within the program would be treated confidentially and in compliance with Organic Law 3/2018 on the Protection of Personal Data and Guarantee of Digital Rights. The data collected were used exclusively for educational and research purposes, ensuring anonymity and the protection of personal information at all times. In addition, supervision and emotional support spaces were provided to accompany the training process and to address any potential effects arising from the simulated clinical practice, thereby ensuring an ethically sound participation and safeguarding students' well-being.

At the beginning of each course, the project was presented to the students, accompanied by a pre-test evaluation regarding their perceived professional competencies. However, it was not until the end of February that the formal organization of the working groups commenced, and the relevant instructions were reiterated. It was deemed necessary for students to have reached a sufficient level of theoretical progress in the courses *Framework* and *Models*, ensuring they possessed the conceptual tools required to approach the practical work with a solid foundation. Consequently, the implementation of the project extended over approximately three months (February to May).

Each session with the patient lasted between 25 and 35 minutes. Prior to each session, the assigned students presented the outline and objectives of the intervention. Faculty members from each course accompanied and supervised the students during their respective classes. Although neither instructor was present at every session, they maintained ongoing communication to coordinate the process and ensure continuity in case monitoring. Unlike conventional classes, these sessions were structured so that the first 30 min were dedicated to the clinical session, while the remaining time was allocated to supervision and accompaniment. This latter phase aimed to analyze the theoretical-practical aspects of the case and explore the students' emotional responses to the clinical experience.

During the supervision phase, constructive feedback was provided, highlighting strengths and areas for improvement, with the objective of fostering professional development and promoting the theoretical-practical integration of the course content. Finally, upon completion of the five sessions, post-project evaluations were conducted to assess the progress and development of the competencies acquired throughout the project.

### Data analysis

Prior to inferential testing, an exploratory data analysis (EDA) was conducted to examine the distribution of the variables, detect potential outliers, and check for missing data. Descriptive statistics (means, standard deviations, skewness, and kurtosis) were revised for all competency dimensions. Skewness and kurtosis values were within acceptable limits, suggesting an approximately normal distribution.

Although the Shapiro-Wilk test indicated mild deviations from normality in some variables, visual inspection of histograms and Q–Q plots, together with the sample size (*n* = 135), supported the assumption of approximate normality. According to Prado Prado et al. (2015), parametric tests are robust to slight violations of normality when the sample size is moderate or large. Therefore, paired-samples *t*-tests were used to compare pre- and post-intervention scores.

Effect sizes (Cohen's d) were calculated to assess the magnitude and practical relevance of the observed changes, emphasizing effect size interpretation over mere statistical significance. All analyses were conducted using IBM SPSS Statistics (version 30.0).

## Results

To assess the effectiveness of the educational innovation program implemented over two academic years, a comparison of pre-test and post-test scores from the employed scales was carried out. Paired-samples t-tests were applied to compare pre- and post-intervention results for each evaluated competency. The descriptive pre–post results of the evaluated dimensions are presented in Table 3.

**Table 3:** Descriptives of the dimensions of the intervention pre and post.

**Competencies**	***M (SD)***	
	**Pre**	**Post**
Autonomous learning	18.33 (2.80)	19.96 (2.56)
Knowledge application	14.39 (2.08)	15.28 (1.88)
Teamwork	15.09 (1.99)	15.39 (1.99)
Argumentation	18.75 (3.19)	20.15 (2.56)
Problem solving	18.62 (2.59)	19.89 (2.63)
Analysis of information	19.08 (2.71)	19.99 (2.46)
Communication	19.19 (2.80)	20.30 (2.42)
Ethical awareness	18.84 (3.16)	20.39 (2.41)
Creativity	13.41 (2.30)	14.73 (2.06)
Evaluation	18.10 (2.94)	20.31 (2.44)

*M* and *SD* represent the mean and standard deviation of the descriptive scores for each dimension.

In addition, considering the above, pre-post data for both academic years (2023–2024 and 2024–2025) (see Table 4). The results revealed improvements in most evaluated competencies: autonomous learning, knowledge application, argumentation, problem-solving, information analysis, communication, ethical awareness, creativity, evaluation with the exception of the teamwork dimension, which showed minimal change.

**Table 4:** Pre-post mean comparison according to paired samples *t*-test.

**Competencies**	**Intervention**	**Descriptive statistics**		**Paired samples** ***t*****-test**	
		*M*	*SD*	*p*	*d* *de* *Cohen*
Autonomous learning	Pre-post	−1.63	2.59	< 0.001	−0.62
Knowledge application	Pre-post	−0.88	2.17	< 0.001	−0.41
Teamwork	Pre-post	−0.29	2.28	0.134	-
Argumentation	Pre-post	−1.40	2.97	< 0.001	−0.47
Problem solving	Pre-post	−1.26	2.76	< 0.001	−0.45
Analysis of information	Pre-post	−0.90	2.82	< 0.001	−0.32
Communication	Pre-post	−1.11	2.61	< 0.001	−0.42
Ethical awareness	Pre-post	−1.54	3.42	< 0.001	−0.44
Creativity	Pre-post	−1.31	2.40	< 0.001	−0.54
Evaluation	Pre-post	−2.21	2.83	< 0.001	−0.78

*M* and *SD* represent the mean and standard deviation of the difference between post- and pre-intervention scores.

Effect sizes were calculated using Cohen's *d* to estimate the magnitude of change and to interpret the practical significance of the results. According to conventional benchmarks (Cohen, 1988) the competencies of autonomous learning, argumentation, problem-solving, evaluation, and creativity showed large effects, whereas knowledge application, information analysis, communication and ethical awareness demonstrated medium effects.

## Discussion

In the current university landscape, the training of psychology professionals requires a comprehensive approach that integrates innovative methodologies aimed at the practical development of essential competencies. Unlike traditional methods, innovative educational approaches adopt a student-centered model, recognizing students as active agents in their own learning process, stimulating their curiosity and intrinsic motivation (Crespí et al., 2022; Herrada and Baños, 2018). In this regard, the present study aimed to evaluate the effectiveness of a teaching innovation program based on simulated contexts under the accompaniment and supervision of a qualified instructor, designed to strengthen competencies essential for future professional practice.

Following the implementation of the program, improvements were observed in most of the evaluated competencies, while teamwork showed only minimal change. Although several differences reached statistical significance, the interpretation of the findings was based primarily on effect sizes and the practical meaning of the observed changes rather (Campbell et al., 2010). These findings align with previous studies such as Gil-Galván et al. (2020), which highlight the value of innovative teaching methodologies, such as problem-based learning, where students appreciate activities that help them connect theoretical knowledge with real-life applications (Hebles et al., 2019). Therefore, it is essential to move away from traditional teacher-centered methods and update higher education toward new learning processes focused on practical student experiences.

Improvements in competencies such as argumentation, problem-solving, evaluation, and creativity suggest that the program's design, characterized by an active approach and continuous teacher accompaniment, served as a facilitator for transferring these skills to applied contexts. In particular, clinical supervision provided a space in which students could articulate the challenges encountered and receive guidance on how to address them, enhancing their professional development (Yung, 2023). Moreover, the active role involved in managing the clinical case required students to make therapeutic decisions that directly fostered some of the evaluated competencies, such as communication, argumentation, or information analysis. The effect sizes obtained in these competencies ranged from medium to large, suggesting that the program contributed meaningfully to students' professional skill development despite the modest absolute changes in mean scores.

These findings are especially relevant as they underscore the practical impact of the program on developing skills related to evidence-based decision-making, critical analysis, and adaptability to complex clinical contexts. They also reinforce the idea that expert supervision enhances learning and fosters the development of key competencies for future professional practice.

Another relevant aspect to highlight in the present study is that the instruments employed have shown strong psychometric qualities in prior research, supporting the reliability of the results. Still, the modest mean differences observed could partly be due to measurement characteristics, such as limited variance or ceiling effects, rather than substantial educational gains. This aspect should be carefully considered in future studies, which could incorporate additional or more sensitive measures to better capture changes in competence development.

### Practical implications

These results support the incorporation of active methodologies in university classrooms as a strategy to enhance the development of transversal competencies essential for the professional practice of psychology and other health-related disciplines (Crespí et al., 2022; Yeung et al., 2024). In this context, problem-based learning and cooperative learning emerge as effective and practical methodologies that require mature communication, interdependence, and motivation to work toward shared goals (Herrada and Baños, 2018).

Although cooperative and group-based learning methodologies have proven effective in developing collaboration and coordination skills (Hebles et al., 2019; Martínez-Clares and González-Morga, 2018), the absence of significant change in the teamwork competency may be due to contextual factors or the individual nature of the proposed activities. Additionally, the time allocated to the intervention may not have been sufficient to generate an observable impact on this competency, which by nature requires continuity, collaborative experiences, and time for consolidation. On the other hand, the pre-intervention planning reflected a more independent design, supported by the significant improvement in the autonomous learning competency (Yeung et al., 2024). This reality may have influenced cooperation and team coordination, thus affecting the intervention's effectiveness in strengthening teamwork.

Additionally, recent research indicates that the development of collaborative competencies requires not only time but also a pedagogical structure that fosters positive interdependence, individual accountability, and promotive interaction among group members (Mendo-Lázaro et al., 2018). The absence of these conditions may limit the effectiveness of group activities in strengthening teamwork. Likewise, Lacerenza et al. (2018) emphasize that short-term interventions tend to yield limited effects on group competencies. Furthermore, factors such as individual motivation or the perceived value of the task have also been found to significantly influence the quality of group interactions (Lacerenza et al., 2018; Li et al., 2025), which may have affected the results observed in this competency.

### Study limitations

Although the study yielded positive results, confirming the importance of involving students in practical activities closely aligned with clinical reality through innovative methodologies that enhance their perception of competencies, some limitations should be acknowledged. Firstly, given the quasi-experimental design, the absence of randomization in participant assignment may have introduced bias related to uncontrolled variables, such as motivation levels or previous experiences. Nonetheless, this methodological choice was guided by practical and ethical considerations inherent to educational settings, prioritizing the feasibility of the intervention in real classroom environments. Furthermore, the lack of a control group prevents the exclusion of external factors, such as parallel academic influences or students' natural developmental progress, as potential contributors to the observed changes. In this regard, the small but consistent improvements observed could also reflect students' normal progression or measurement limitations rather than the exclusive effect of the program. However, the pre-post quasi-experimental design and the use of robust statistical analyses aimed to mitigate this potential bias. Finally, the limited time available to conduct all sessions constrained the scope of the intervention, and a greater number of sessions would have been beneficial. Future research should include control groups, longitudinal follow-ups, and complementary qualitative data to explore students' perceptions of competence development more deeply and to understand the processes underlying the observed improvements.

## Conclusion

The findings of this study confirm the effectiveness of the intervention, demonstrating significant improvements in the evaluated competencies by providing a hands-on experience closely aligned with real-world clinical practice. The methodology enabled the integration of theoretical and practical work alongside clinical supervision, fostering the development of essential skills for students' future professional roles.

In this regard, future research should explore how the number of sessions influences the consolidation of acquired competencies, assessing whether an increased volume of practical experiences significantly enhances therapeutic competencies. Additionally, longitudinal studies evaluating the long-term effectiveness of these methodologies would be valuable. Such research could explore the extent to which competencies trained in simulated settings translate into effective clinical practice in real-world environments during the early years of professional work.

## Data Availability

The raw data supporting the conclusions of this article will be made available by the authors, without undue reservation.
